# Calcium Gluconate in Phosphate Buffered Saline Increases Gene Delivery with Adenovirus Type 5

**DOI:** 10.1371/journal.pone.0013103

**Published:** 2010-09-30

**Authors:** Marko T. Ahonen, Iulia Diaconu, Sari Pesonen, Anna Kanerva, Marc Baumann, Suvi T. Parviainen, Brad Spiller, Vincenzo Cerullo, Akseli Hemminki

**Affiliations:** 1 Cancer Gene Therapy Group, Molecular Cancer Biology Program and Transplantation Laboratory and Haartman Institute and Finnish Institute for Molecular Medicine, University of Helsinki, Helsinki, Finland; 2 HUSLAB, Helsinki University Central Hospital, Helsinki, Finland; 3 Department of Obstetrics and Gynecology, Helsinki University Central Hospital, Helsinki, Finland; 4 Protein Chemistry Unit, Institute of Biomedicine, University of Helsinki, Helsinki, Finland; 5 Department of Child Health, School of Medicine, Cardiff University, Cardiff, United Kingdom; Auburn University, United States of America

## Abstract

**Background:**

Adenoviruses are attractive vectors for gene therapy because of their stability *in vivo* and the possibility of production at high titers. Despite exciting preclinical data with various approaches, there are only a few examples of clear efficacy in clinical trials. Effective gene delivery to target cells remains the key variable determining efficacy and thus enhanced transduction methods are important.

**Methods/Results:**

We found that heated serum could enhance adenovirus 5 mediated gene delivery up to twentyfold. A new protein-level interaction was found between fiber knob and serum transthyretin, but this was not responsible for the observed effect. Instead, we found that heating caused the calcium and phosphate present in the serum mix to precipitate, and this was responsible for enhanced gene delivery. This finding could have relevance for designing preclinical experiments with adenoviruses, since calcium and phosphate are present in many solutions. To translate this into an approach potentially testable in patients, we used calcium gluconate in phosphate buffered saline, both of which are clinically approved, to increase adenoviral gene transfer up to 300-fold *in vitro*. Gene transfer was increased with or without heating and in a manner independent from the coxsackie-adenovirus receptor. *In vivo*, in mouse studies, gene delivery was increased 2-, 110-, 12- and 13-fold to tumors, lungs, heart and liver and did not result in increased pro-inflammatory cytokine induction. Antitumor efficacy of a replication competent virus was also increased significantly.

**Conclusion:**

In summary, adenoviral gene transfer and antitumor efficacy can be enhanced by calcium gluconate in phosphate buffered saline.

## Introduction

The success of gene therapy is dependent on the use of an efficient gene transfer system to allow the expression of the therapeutic gene in the target organ, tissue or cell. Adenoviruses are one of the most popular vectors because of their stability in vivo and the possibility of production of high titers [1]–[3]. Despite exciting preclinical data with various delivery methods, there are only few examples of clear efficacy in clinical trials and all of these trials have employed local delivery [4]–[9].

In contrast, systemic administration of high doses of adenoviruses can result in toxicity characterized by a pro-inflammatory cytokine storm, intravascular disseminated coagulopathy and organ failure [10], [11]. Hence, identification of new approaches that can increase gene delivery without enhanced toxicity remains important.

Classically, the cellular entry of the most common adenovirus gene therapy vector, serotype 5 (Ad5), initiates by binding of the fiber knob to the primary receptor, the coxsackie-adenovirus receptor (CAR) [12]. This interaction has been suggested to be an important rate-limiting step for gene transfer, *i.e.*, lack of CAR could make target tissues refractory. [13]–[16]. It has been also shown that certain blood factors can interact with adenovirus [17], [18].

In one of our previous studies, we anecdotally discovered that heated mouse serum can enhance adenoviral gene delivery to cancer cells in vitro [19]. In this manuscript, we sought to confirm the finding in clinically meaningful substrates and then unravel the mechanism behind it. Finally, we applied the mechanism for enhancing adenoviral gene transfer to tumor tissue and normal organs. Interestingly, we discovered that calcium and phosphate were responsible for the phenomenon. Therefore, our findings extend a classic in vitro technique [20] into in vivo benefits.

## Materials and Methods

### Ethics statement

All animal experiments were approved by the Experimental Animal Committee of the University of Helsinki and the Provincial Government of Southern Finland (ESLH-2008-01986).

### Cell lines and viruses

Cancer cell lines used in this study included human breast cancer cell line M4A4-LM, [21], chinese hamster ovary cell lines CHO (with or without human CAR transgene) [22] . Lung adenocarcinoma cells A549 were bought from ATCC (Manassas, VA USA). All cell lines were maintained in the recommended conditions.

Replication deficient adenovirus: Ad5(gl) encoding luciferase and green fluorescent protein and were propagated on 293 cells and purified on cesium chloride gradients as described [23]. The viral particle (VP) concentration was determined at 260 nm, and standard plaque assay on 293 cells was performed to determine infectious particles.

Ad300wt is a replication competent wild type adenovirus 5 acquired from ATCC.

### *In vitro* gene transfer assay

Different concentrations of mouse serum (Sera Laboratories International, United Kingdom), vitamin B mixture (Orion Pharma, Finland), purified vitamin Bs (Sigma Aldrich), Dulbecco's modified Eagle's medium (DMEM) (Sigma Aldrich), calcium chloride (CaCl_2_) (Sigma Aldrich), EDTA (Sigma Aldrich) or calcium gluconate (CaGl) (B Braun, Germany) were mixed with phosphate buffer solution (PBS). Mixtures were heated to 80°C for 30 min and cooled down to room temperature (RT). Mixtures were vortexed and supernatant was collected. The pH was adjusted to 7.4 with 1M NaOH. Viruses at 100vp/cell were added to the mixtures and incubated for 30 min at room temperature.

Cell lines seeded in 24 well plates in quadruplicates at 1×10^4^ cells/well were incubated with Ad5(gl) in the mixtures described above for 30min. Infection media was replaced with fresh growth media containing 10% FCS and cell monolayers were incubated for 48h . Cell lysates were analyzed using luciferase assay (E1500, Promega, WI, USA).

A549 cell line monolayers were infected with either Ad5(gl) and CaGl or Ad5(gl) alone for 30minutes. Infection media was replaced and plates were visualized for GFP expression 24 hours post infection.Pictures were taken at 10× magnification with an axiovert 200 microscope.

### *In vitro* replication assay

Calcium gluconate mixtures were prepared as described for in vitro gene transfer assay. Replicating Ad300wt at 10VP/cell was added to the mixtures and incubated for 30 min at room temperature. A459 cells were seeded in 12 well plates in quadruplicates at 15×10^4^ cells/well were incubated with virus in mixtures described above for 30 min. Infection media was replaced with fresh growth media containing 10% FCS and cell monolayers were incubated for 2h, 6h, 18h, 30h, 42h, 54 or 66h. Cell lysates were analyzed using quantitative PCR (Lightcycler, Roche, Mannheim, Germany) as described [24].

### Mass spectrometry (MS) analysis

Recombinant Ad5 knob proteins were bound to magnetic beads (Qiagen, Germany). Heated or unheated serum was added and the mixture was incubated for 2h at 4°C. The complexes were washed with ice-cold PBS five times. Knob-interacting proteins were eluted with 8M urea. The denaturated proteins were run on a 10% SDS-PAGE gel, silver stained (GE healthcare, United Kingdom), concentrated with ZipTip RP C-18 (Millipore, Bedford, MA, USA) and further analyzed by a Bruker Daltonics (Bremen, Germany) Autoflex III mass spectrometer operated in the linear positive ion mode (large proteins) or the reflector mode (smaller peptides) [25].

### Fluorescence-activated cell sorting array

Mouse interleukin 6 (IL-6), IL 12p70 and TNF-α were analyzed with a BD cytokine multiplex bead array system (BD FACSArray; BD Biosciences, San Jose, CA).

### Animal experiments

Immune-competent ICR mice (Taconic, Ejby, Denmark) were quarantined for 2 weeks. Mice were housed in plastic cages with wire mesh covers in a room with a 12-h light–dark cycle with 50±10% relative humidity at 24±1°C. In the intravenous experiment, c57bl mice were injected with 1×10^9^ VP or 1×10^10^ VP Ad5(gl) (low or high dose group) mixed with CaGl (4.5mg/ml). After 6h, serum was collected for cytokine analysis. After 48h mice were killed and organs were collected and stored at −80°C. Organs were homogenized and lysed with Cell Culture Lysis Buffer (Promega Corporation, Madison, WI, USA, Nr. E153A), freeze-thawed three times, and supernatants were analyzed with FluoStar Optima luminometer. Results were normalized to protein content by DC Protein Assay (Bio-Rad, Hercules, Manassas, CA, USA).

In the intratumoral experiment, nude mice were injected orthotopically into the left and right uppermost mammary fat pad with 2×10^6^ M4A4-LM3 cells and a tumor was allowed to develop [21]. When tumors reached approximately 0.5 cm, mice were randomized into groups and injected with 1×10^7^ VP/tumor of Ad5(gl) with CaGl (1.2mg/ml) or with PBS (*n* = 8 tumors/group). Viruses were injected intratumorally in a total volume of 50 µl. After 24h mice were killed and tumors were collected and stored at −80°C. Tumors were homogenized, lysed, analyzed and normalized as described above.

For the anti-tumor efficacy experiment, tumors were grown in nude mice as above. When tumors reached approximately 0.5cm, mice were randomized into groups and injected with 1×10^7^ VP/tumor of replicating Ad300wt with heated CaGl (1.2mg/ml) or with PBS (*n* = 8 tumors/group). Viruses were injected intratumorally in a total volume of 50ìl. Size of the tumors were measured every other day.

### *In vivo* imaging

4.5 mg of D-Luciferin (Promega, Madison, WI, USA) in 100 µl 0% Roswell Park Memorial Institute (RPMI) growth medium was injected intraperitoneally. After 10 min, images were captured with the IVIS imaging system series 100 using Living Image v2.5 software (Xenogen, Alameda, CA, USA) and photon emission values were calculated.

### Statistical analyses

To compare differences between groups, two-tailed student's t-test was used and a *P*-value of <0.05 was considered significant.

## Results

### Gene delivery with adenovirus 5 (Ad5) is increased in the presence of heated serum

**Figure 1:**
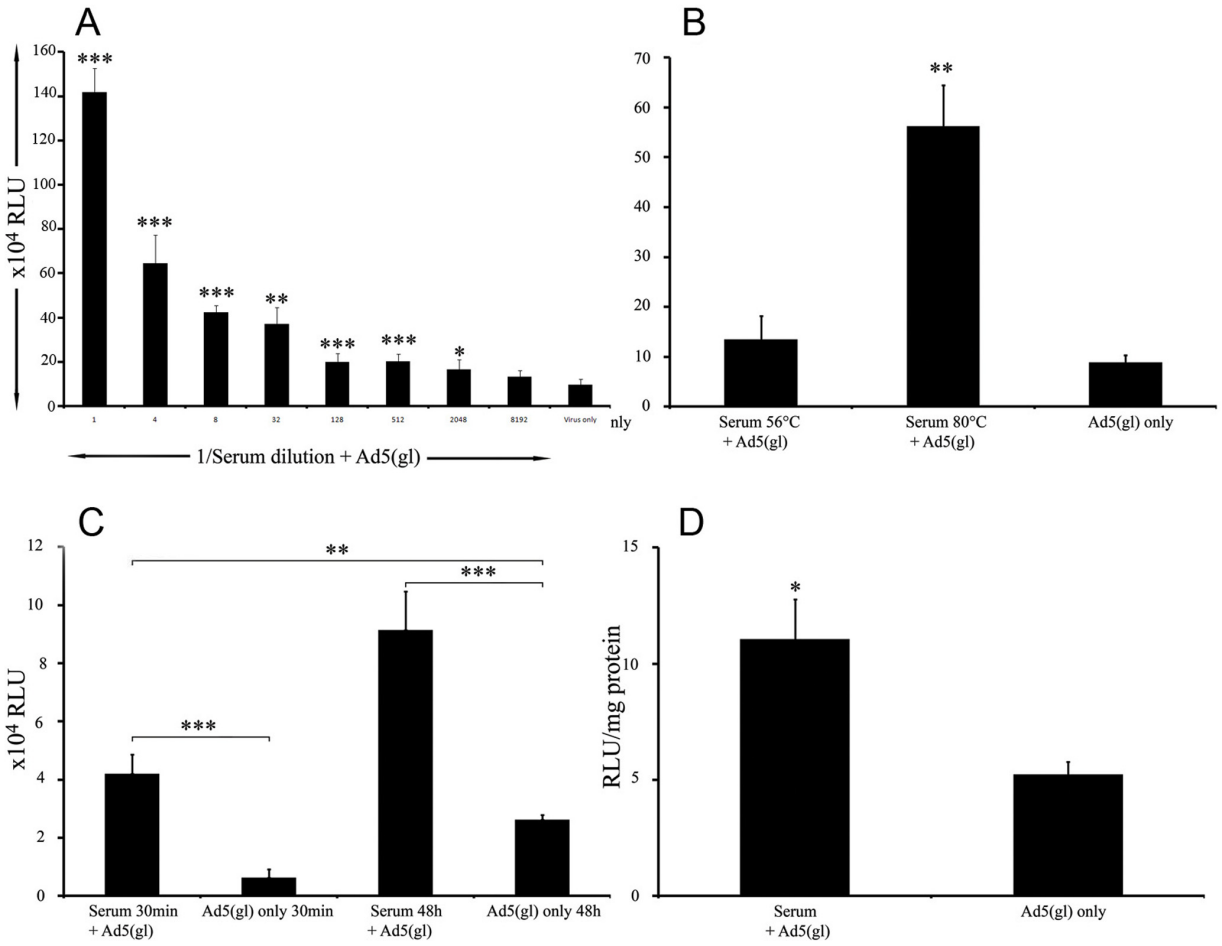
Heated mouse serum increases transgene expression of Ad5(gl) *in vitro* and *in vivo*. a) A549 cells were infected with replication-deficient luciferase expressing Ad5(gl) at a concentration of 100VP/cell with or without heated mouse serum. b) Different heating temperatures of mouse serum (56°C for 1h and 80°C for 30min) and c) different infection periods (30 minutes and 48 hours (h) with or without heated mouse serum. d) Nude mice with M4A4-LM3 mammary fat pad tumors were injected intratumorally with a Ad5(gl) at a dose of 1×10^7^ VP/tumor (n = 4 mice/group). Tumors were collected 48h following injection, lyzed for luciferase assay and normalized to protein content. Luciferase activity was measured as relative light units (RLU) 48 hours after infection. *,P<0.05; **,P<0.01 and ***,P<0.001.

In an earlier study [19] we found that serum from non-immunized mice heated to 65°C (to inactivate complement) increased transgene expression *in vitro*. To confirm this finding we analyzed transgene expression of Ad5(gl) with serial dilutions of heated serum on UT-SCC29 (not shown) and A549 cells (Fig. 1a). We observed up to 20 fold increased transgene expression in cells infected with mixture of Ad5 with the highest concentration of serum compared to cells infected with virus diluted in PBS.

To further characterize the observation we performed a series of experiments where cells were infected with a mixture of Ad5 and heat-inactivated serum at different temperatures. We observed that when serum was subjected to 56°C for 30min (another commonly used protocol to heat-inactivate complement) transgene expression was not affected but when heated to 80°C a significant increased in transgene expression was seen (Fig. 1b). Further, we analyzed different lengths of incubation of virus mixed with serum. At both 30min and 48h we noticed a significant increase of transgene expression when compared with virus only (Fig. 1c).

To assess whether this observation was translatable into more clinically relevant *in vivo* results, mice bearing subcutaneous tumors were injected intratumorally either with virus or with virus mixed with heated serum. Significantly higher transgene expression was observed in the group of mice treated with Ad5(gl) mixed with heated serum (Fig. 1d). In conclusion, we found that heated serum can enhance gene delivery of adenovirus in vitro and in vivo.

### Mouse transthyretin (TTR) binds to Ad5 knob

**Figure 2:**
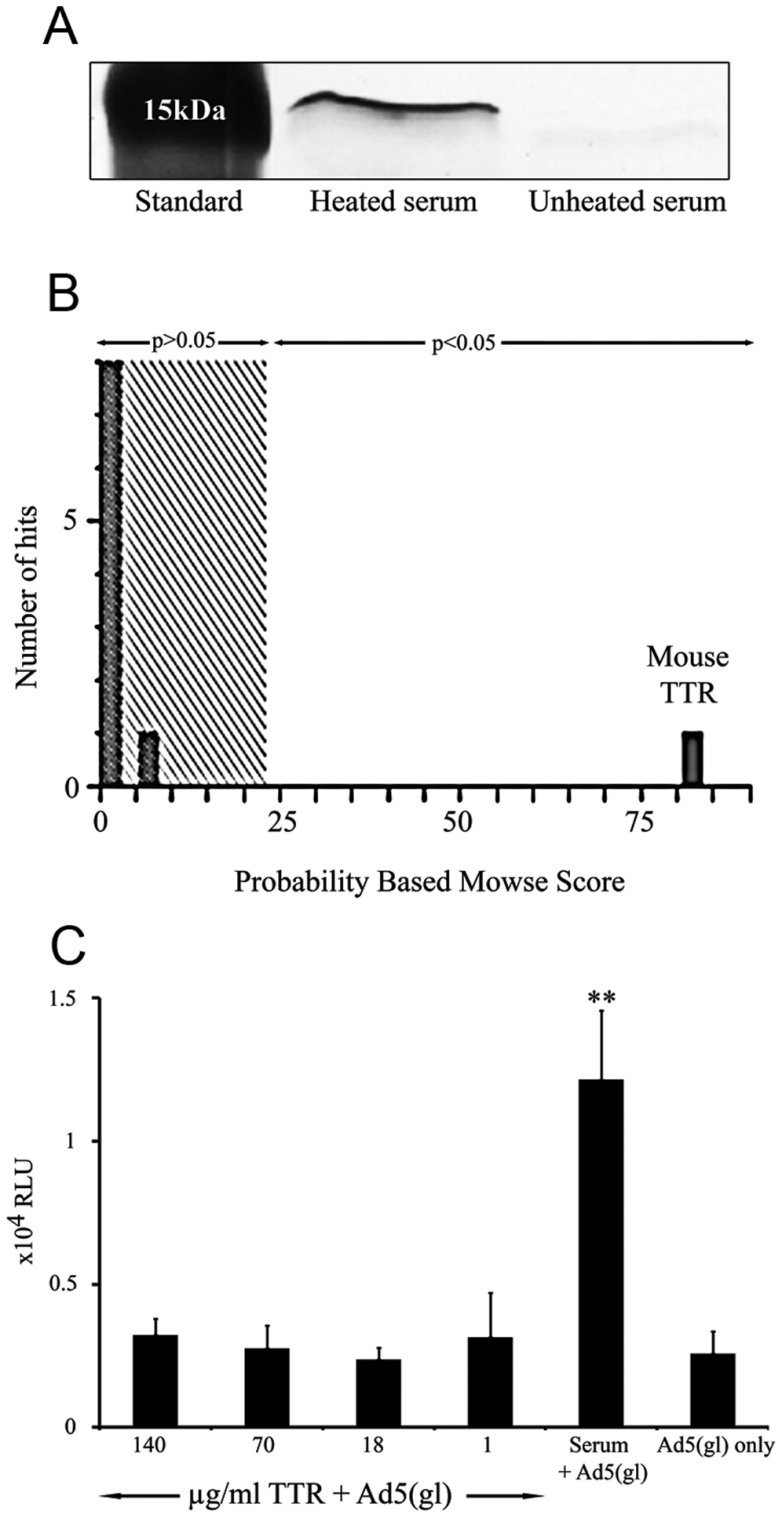
Identification of Ad5 knob binding proteins in heated mouse serum. a) Silver stained gel showing molecular-weight marker and proteins probed with Ad5 knob from heated and unheated mouse serum. b) Mass spectrometry analysis of 15kDa protein probed from heated mouse serum identified mouse transthyretin (TTR) as an Ad5 knob binding protein. c) Lung adenocarcinoma A549 cells infected with replication-deficient luciferase expressing Ad5(gl) at a concentration of 100VP/cell with or without different concentrations of heated recombinant TTR or heated mouse serum. TTR did not increase gene transfer. Luciferase activity was measured as relative light units (RLU) 48 hours after infection. **,P<0.01.

We hypothesized that the factor interacting with Ad5 and mediating enhanced delivery would be a protein. Therefore, heated and unheated mouse serum was precipitated with Ad5 fiber knob domains and analyzed by tandem mass spectrometry. A protein of the size of 15kDA was identified in the fraction corresponding to heated serum incubated with fiber knob; such a protein was not present in the fraction corresponding to un-heated serum preincubated with fiber knob (Fig. 2a). The identified protein was analyzed by mass spectrometry and identified as monomeric mouse transthyretin (TTR). TTR is a tetrameric protein composed of identical subunits having a predominantly β-sheet structure [26]. TTR is found in serum and cerebrospinal fluid of mammals and it functions as a secondary carrier for thyroid hormone thyroxin and for retinol-binding protein [27]. It is a heat-resistant protein that only partially denaturates after heating and almost completely regains its normal form after re-cooling [*28*].

After establishing the interaction of Ad5 with TTR, we investigated whether this was responsible for enhanced transduction. A549 cells infected with or without purified mouse TTR resulted in no change in gene delivery (Fig. 2c). We concluded that even though there is a physical interaction between the knob domain of the adenovirus 5 fiber knob and TTR, this interaction does not influence adenovirus cell entry.

### D-Pantothenic acid hemicalcium salt (vitamin B5) contained in cell growth media increases Ad5 gene delivery

**Figure 3:**
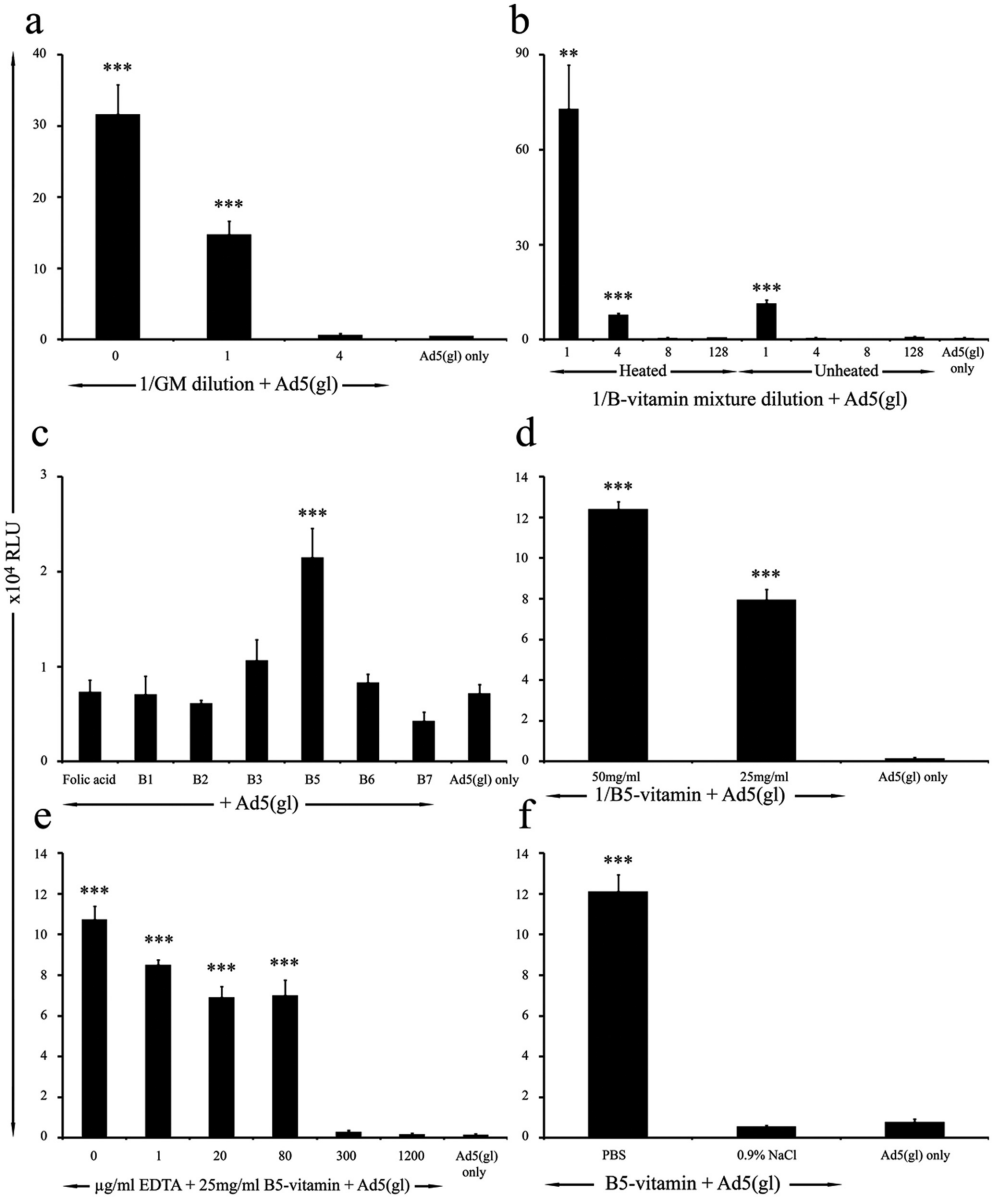
Heated D-Pantothenic acid hemicalcium salt (vitamin B5), present in cell growth media, increases transgene expression of Ad5(gl) *in vitro*. A549 cells infected with replication-deficient luciferase expressing Ad5(gl) at a concentration of 100VP/cell with or without a) dilutions of heated growth media, b) heated and unheated dilutions of vitamin B mixture, c) heated vitamin B's (4µg/ml of folic acid, 4µg/ml of vitamin B1, 0.4µg/ml of vitamin B2, 4µg/ml of vitamin B3, 4µg/ml of vitamin B5, 4µg/ml of vitamin B6 and 7.2µg/ml of vitamin B8), d) different concentrations of heated vitamin B5, e) 25mg/ml of heated vitamin B5 with different concentrations of calcium-chelating ethylenediaminetetraacetic acid (EDTA) indicate calcium as the relevant component in vitamin B5. f) 25mg/ml of heated vitamin B5 in PBS or in physiological saline solution (0.9% NaCl) demonstrate the requirement for phosphate for enhanced gene delivery. Luciferase activity was measured as relative light units (RLU) 48 hours after infection. **,P<0.01 and ***,P<0.001.

Because TTR was the only candidate protein identified in mass spectrometry, we hypothesized next that the causative molecule would not be a protein. Moreover, we performed experiments to clarify if the putative gene transfer increasing agent was present in the serum of mice or the growth medium used as a diluent in all experiments. Up to 60 times increased transgene expression was seen with heated growth media compared to virus only (Fig. 3a). As heated serum alone had no major influence on transgene delivery, we concluded that the effect had been in fact mediated by the growth medium used as diluent.

One of the main constituents of growth media are B vitamins. Thus, we analyzed Ad5(gl) transgene delivery with serial dilutions of heated and unheated vitamin B mixture. Up to 140 and 20 times (heated and unheated vitamin B mixture) increased transgene expression was seen (Fig. 3b). When we tested the different B vitamins individually, only vitamin B5 increased transgene expression (Fig. 3c and d).

Vitamin B5 is conjugated with calcium which dissolves when mixed to any solvent [29]. The solvent used in these experiments was PBS. Interestingly, it is well known that calcium and phosphate form calcium phosphate (CaPi) precipitates which can increase transgene expression of adenoviruses [30]–[36]. EDTA is a chelating agent that can bind to calcium and block it. When we combined heated vitamin B5 in PBS with EDTA, the increase in transgene expression was ablated in a dose dependent manner (Fig. 3e). Also, an absence of increase in transgene expression was seen when mixing vitamin B5 to saline solution (0.9% NaCl) which does not contain any phosphate (Fig. 3f) suggesting that the effect required both calcium and phosphate. As suggested previously [30], the effect may be mediated through CaPi precipitates, since we visually detected precipitates in the test tube and more precipitates were seen when heating was applied.

### Calcium gluconate (CaGl) enhances adenovirus transduction *in vitro*

**Figure 4:**
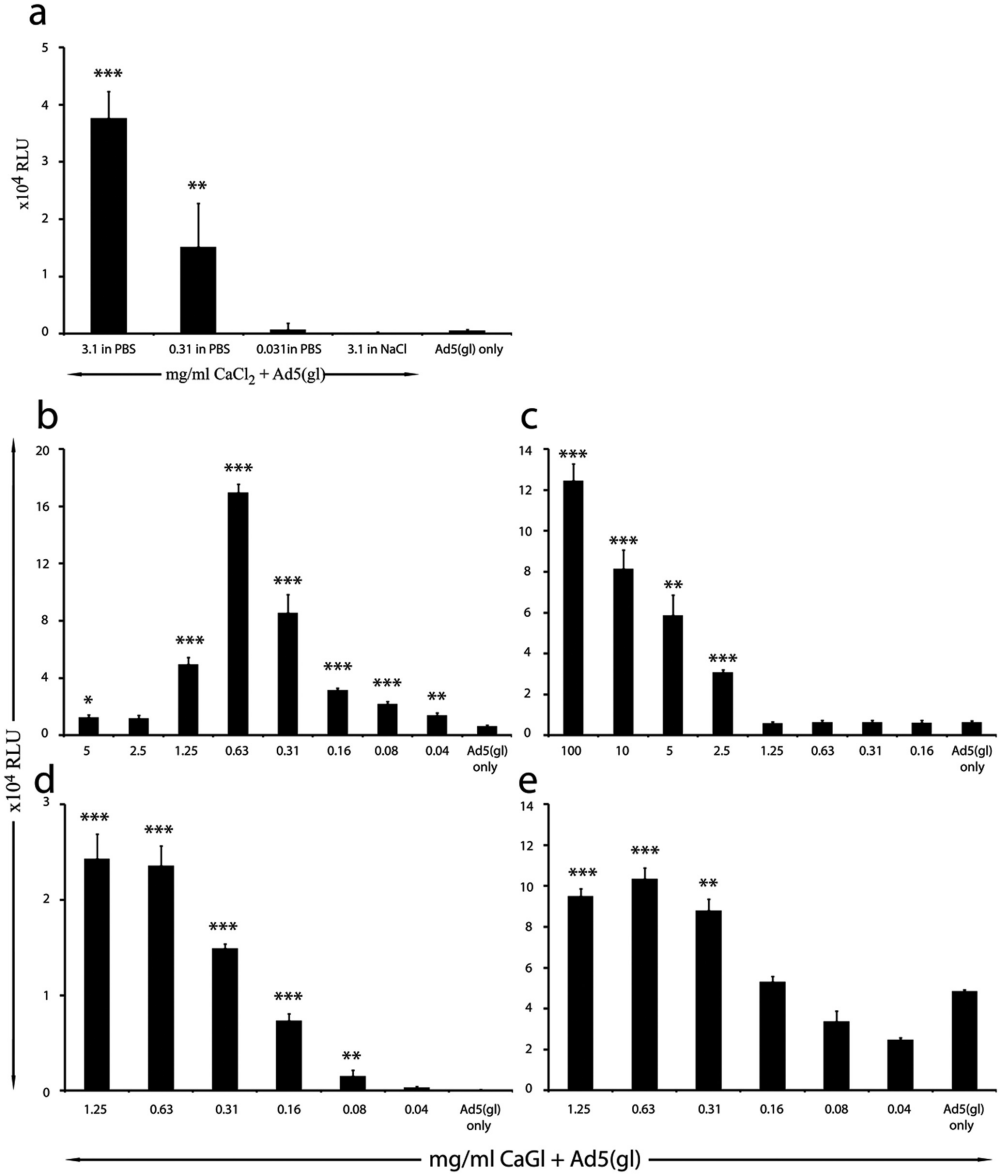
Calcium gluconate (CaGl) increases transgene expression of Ad5(gl) *in vitro*. a) Lung adenocarcinoma A549 cells infected with replication-deficient luciferase expressing Ad5(gl) at a concentration of 100VP/cell with different concentrations of calcium chloride (CaCl_2_) mixed to PBS or 3.1mg/ml of CaCl_2_ mixed to physiological saline (0.9% NaCl). b) A549 Cells infected with Ad5(gl) at 100VP/cell with different dilutions of heated or c) unheated CaGl. CAR negative Chinese hamster ovary (CHO) cells and CHO cells expressing human CAR were infected with Ad5(gl) at a concentration of 100VP/cell with or without CaGl, demonstrating that the effect was not CAR-dependent d) CAR negative CHO cells and e) CHO with transgenic CAR. Luciferase activity was measured as relative light units (RLU) 48 hours after infection.*,P<0.05; **,P<0.01 and ***,P<0.001.

CaPi precipitates have been reported to increase transgene expression of adenovirus in vitro as well as in vivo [30], [33], [35]. However, there is no clinically approved approach allowing human use of this information. Therefore, we tested if calcium gluconate (CaGl) could be combined with PBS for results similar to what were seen with serum and vitamin B5. CaGl increased gene delivery up to 75-fold when combined to PBS but not when combined to NaCl (Fig. 4a). CaGl is globally approved for treatment of hypocalcaemia [37], [38] while PBS is also widely used in humans; typically when an isotonic and non-toxic solution is needed [39]–[41]. To assess dose response, we analyzed serial dilutions of heated CaGl and found that intermediate doses gave the best results (Fig. 4b). Importantly, also unheated CaGl increased gene delivery up to 20 fold (Fig. 4c) CaGl with Ad5(gl), which might perhaps facilitate clinical testing.

To investigate if the effect was CAR related, we studied CHO cells which do not express endogenous CAR. As a control we used CAR transfected CHO cells which stably express CAR [22]. The former are known to be resistant to adenovirus, as confirmed here (Fig. 4d), whereas the latter are susceptible (Fig. 4e). While CaGl increased gene delivery in both cell lines, the effect was more dramatic in cells lacking CAR (Fig. 4d). This suggested that the calcium-phosphate mediated effect was independent of CAR.

### CaGl enhances adenovirus transduction *in vivo*

**Figure 5:**
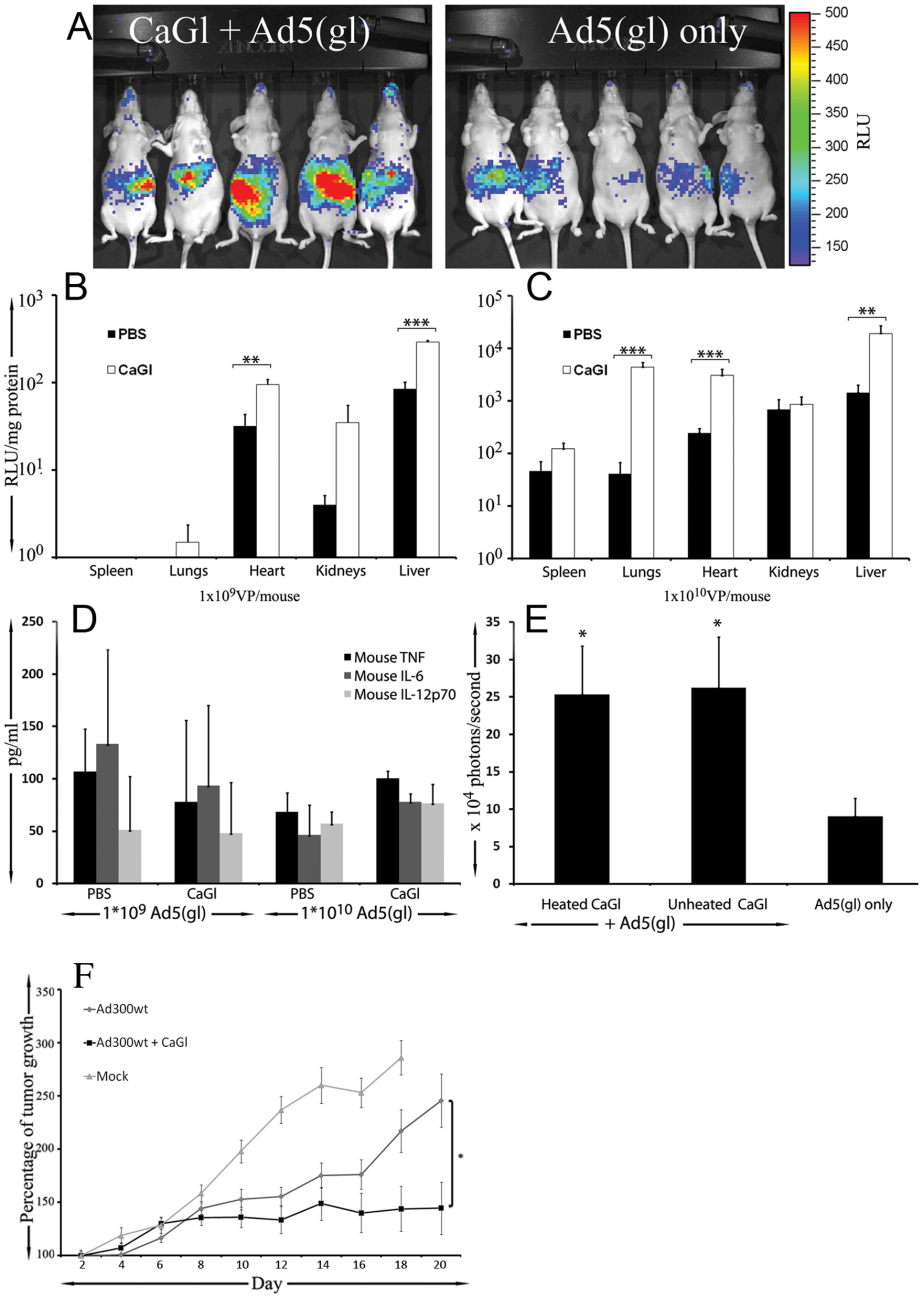
CaGl enhances adenoviral gene delivery to tumors, liver, lung and heart and increases anti-tumor efficacy without an increase in pro-inflammatory cytokines *in vivo*. a) Photon emission at 24 h from mice injected intravenously with replication-deficient luciferase expressing Ad5(gl) at a dose of 5×10^9^ VP/mouse with or without 4.5mg of calcium gluconate (CaGl) (n = 4 mice per group). Increased liver transduction is seen in whole body imaging. Organs were collected from mice were injected intravenously with Ad5(gl) at a dose of b) 1×10^9^ VP/mouse and c) 1×10^10^ VP/mouse (n = 4 mice per group) with or without 4.5mg of heated CaGl. An increase in gene transfer was seen in lungs, liver and heart. d) 6 h after virus injection, mouse serum was analyzed for mouse interleukin 6 (IL-6), IL 12p70 and TNF- α. No increase in these adenoviral toxicity markers was seen. e) Nude mice with M4A4-LM3 mammary fat pad tumors were injected intratumorally with Ad5(gl) at a dose of 1×10^7^ VP/tumor (n = 4 mice/group). with or without 1.2mg/mg CaGl. Both heated and unheated CaGl increased gene delivery. f) Nude mice with M4A4-LM3 mammary fat pad tumors were injected intratumorally with replication competent (and therefore lytic) wild type adenovirus type 5 at a dose of 1×10^7^ VP/tumor (n = 5 mice/group). with or without heated CaGl (1.2mg/ml) and followed tumor growth for 20 days. *,P<0.05; **,P<0.01 and ***,P<0.001.

Significantly higher transgene expression, as detected by in vivo imaging, was observed 24h after virus injection in the group of mice treated with Ad5(gl) with CaGl (2.8±0.5 versus 1.3±0.2×10^5^ photons/s, Fig. 5a). The same result was seen when organs were analyzed ex vivo at 48 hours (Fig. 5b–c).

Increases in gene transfer would be most useful if not accompanied by increases in toxicity. Therefore, serum cytokines were measured 6h after virus injection. Interestingly, our analysis showed that there was no increase in proinflammatory cytokines in the combination with CaGl (Fig. 5d).

With regard to cancer treatment, adenovirus is most often delivered intratumorally and this was therefore employed here. Significantly higher transgene expression was observed 24h after injection CaGl with virus when compared to virus only (Fig. 5e). Unheated CaGl was as effective as heated CaGl. CaGl also resulted in significant anti-tumor efficacy when combined with a replication competent virus (Fig. 5f). To summarize the in vivo CaGl experiments, we observed that CaGl treatment enhances adenovirus transduction to both normal organs and tumors without increasing proinflammatory cytokine production.

### CaGl increases the number of cells transduced but not does not affect virus replication

**Figure 6:**
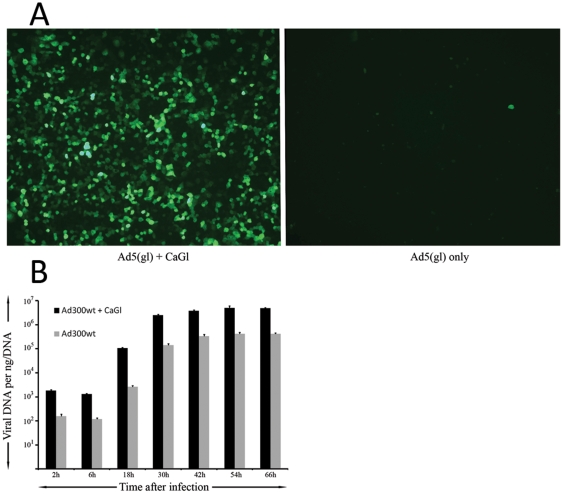
CaGl only affects transduction *per se* and not virus replication. a) A549 cells were infected with replication-deficient luciferase expressing Ad5(gl) at a concentration of 100VP/cell with or without CaGl and imaged after 24h for GFP fluorescence. b) A549 cells were infected with replication competent wild type 5 adenovirus at a concentration of 10VP/cell with or without CaGl and analyzed for viral DNA copy number at different time points.

To investigate if increased transgene expression resulted from a larger number of cells transduced or enhanced transgene expression from the same amount of cells, infection with Ad5(gl) was performed and GFP positive cells were imaged (Fig. 6a). A higher proportion of cells were found GFP positive when CaGl was used.

To assess if CaGl impacted post-transduction steps, cells were infected with a wild type adenovirus with or without CaGl (Fig. 6b). The virus replicated effectively regardless of CaGl which therefore affected only transduction and did not compromise post-entry steps.

## Discussion

Adenovirus-mediated gene therapy holds potential for many diseases, but especially for applications requiring high levels of target tissue transduction. While significant advances in clinical adenoviral gene therapy applications have been made in especially cancer treatment, the clinical translation of adenoviral gene replacement therapy for other diseases has lagged behind. The field remains restrained by innate immune responses inherent to adenovirus, which decreases the therapeutic index of systemic administration of adenovirus particles. Specifically, the efficacy to toxicity ratio of current approaches has not been sufficient to lead to clinical successes heretofore. Thus, development of vector and pharmacologic manipulations, such as suggested here, might contribute to minimizing vector toxicity while maximizing the efficacy of systemic and local adenovirus gene transfer.

Previously it has been shown by Shayakhmetov et al that adenovirus knob can be used to precipitate interacting serum factors and that they are numerous [18]. Many proteins are denaturated by heating but serum is known to have some heat resistant proteins which acquire their native or pseudo-native form after heating [28]. Here we show a previously undescribed interaction between Ad5 knob and mouse serum component transthyretin (TTR). However, TTR did not increase transgene expression. In its naïve form TTR is present in serum as a tetramer. When denatured by high temperature, the monomer had high affinity for adenovirus knob. We believe this finding may have relevance in the context of several diseases *i.e.* senile systemic amyloidosis, familial amyloid polyneuropathy and familial amyloid cardiomyopathy, where high levels of monomeric TTR are present [42]–[44]. It might be worthwhile to study if Ad5 could be used for effective transduction of tissues with high monomeric TTR. Also there is some evidence that ischemic areas of the brain may feature expression of monomeric TTR [45]. This might have relevance for adenovirus biodistribution in treatment of brain diseases such as stroke or glioma.

Since serum proteins precipitating with Ad5 knob did not yield an answer to increased transgene expression we sought to investigate if heating altered other components of the serum/virus mix, including the growth media used for dilution of the serum for normalization of volume. Growth media is a fairly simple solution consisting only of minerals, amino acids and B vitamins. Increased transgene expression was seen with vitamin B mixture and specifically D-Pantothenic acid hemi calcium salt (Vitamin B5), with or without heating. Interestingly, vitamin B5 was also the only tested vitamin that conjugates with soluble calcium. Ethylenediaminetetraacetic acid (EDTA) is known for its ability to chelate metal ions such as calcium [46]. When EDTA was added, the effect of vitamin B5 was ablated (Fig. 3e).

The solvent used in these experiments was PBS. When vitamin B5 was diluted to saline solution that does not contain any phosphate, no increase in transgene delivery was seen (Fig. 3f). These data suggest that calcium and phosphate are necessary for the increased transgene expression seen. Therefore, our initial findings with heated serum led to the well established phenomenon of enhancement of adenovirus transduction with calcium phosphate [30]–[36].

Our results are in accord with previous studies showing that the effect is CAR independent [30], [36]. However, the calcium phosphate technique widely used in the laboratory cannot be applied to humans. Therefore, we sought to extend the approach to calcium and phosphate substrates accepted for human use: CaGl and PBS [37]–[41].

*In vitro* results with CaGl showed up to 300 times increased transgene expression, improved even over vitamin B5 (140 times fold increase). Transgene delivery was increased both with heated and unheated CaGl (Fig. 4b–c) but a stronger effect was seen after heating. Following intravenous injection of CaGl with Ad5 we were able to enhance transduction of many organs (lungs 108, heart 12 and liver 13 fold compared to Ad5 alone, Fig. 5c). No significant increases in cytokine levels were observed (Fig. 5d). This fits well with our current understanding of adenoviral toxicity, whose main determinant might be the adenoviral particles per se being recognized by the innate immune system [11], [47]–[49], while the level of transgene expression may play a smaller role. Alternatively, CaGl+PBS may enhance the transduction of only selected cell populations in organs. For example, in the context of the liver, transduction of hepatocytes might be increased while that of Kupffer cells not. Hepatocytes express transgenes following adenoviral transduction, but Kupffer cells do not [50]. Although cytokines have been proposed as a sensitive marked of adenoviral toxicity [51], it is clear that further studied are needed in this regard. However, it may also be possible the utilization of CaGl could reduce toxicity of an adenoviral treatment if it allows lower virus doses to be used.

As a preliminary disease specific application of the findings, we combined CaGl+PBS with virus for evaluation of the effect on tumor transduction and improved gene delivery was seen and this led also to enhance anti-tumor efficacy when applied in the context of a replication competent adenovirus (Fig. 5e–f). Further toxicity and efficacy studies are needed in other disease specific models. Adenovirus vectors have great potential for the treatment of both hereditary and acquired diseases [52]–[55] and many clinical gene therapy applications require expression of therapeutic transgene at a high level.

In summary, we report that CaGl in PBS can enhance adenoviral gene delivery to several normal organs and also tumors. These findings might have relevance for improving the efficacy versus toxicity ratio of adenoviral gene therapy. Also, awareness of this phenomenon could be important in designing preclinical experiments with adenoviruses, since calcium and phosphate are present in many solutions and might cause misinterpretation of data.
